# COVID-19 Vaccine Hesitancy Among Patients Attending the General Outpatient Clinic in a Tertiary Hospital in Southern Nigeria

**DOI:** 10.7759/cureus.29352

**Published:** 2022-09-20

**Authors:** Samuel O Ilikannu, Omozele M Uwadia, Idotenyin Enyi, Valentine E Idama, Christian C Adugo, Celestina Yusuf, Alexsandra Urhi, Chikodili Ochuba, Omotola Akinade, Gabriel Alugba

**Affiliations:** 1 Obstetrics and Gynecology, Federal Medical Center, Asaba, NGA; 2 Pediatrics and Child Health, Federal Medical Centre, Asaba, NGA; 3 Community Medicine, Federal Medical Centre, Asaba, NGA; 4 Acute Medicine Unit, Chesterfield Royal Hospital, Chesterfield, GBR; 5 Obstetrics and Gynecology, Federal Medical Centre, Asaba, NGA; 6 Internal Medicine, Federal Medical Centre, Asaba, NGA; 7 Family Medicine, Federal Medical Centre, Asaba, NGA; 8 Psychiatry, Federal Neuro-Psychiatric Hospital, Benin City, NGA; 9 Health, Ethics and Society, Maastricht University, Maastricht, NLD; 10 Internal Medicine, General Hospital Ikorodu, Ikorodu, NGA; 11 Internal Medicine, Delta State University, Abraka, NGA

**Keywords:** pandemic, corona virus, vaccine hesitancy, covid-19 vaccine, covid-19

## Abstract

Background

As most of the available studies on acceptance of the coronavirus disease 2019 (COVID-19) vaccine were done prior to the development of a vaccine, this study aimed to determine the current willingness to receive the available COVID-19 vaccines in Nigeria and ascertain factors influencing its acceptance.

Methodology

A cross-sectional descriptive study using a paper-based questionnaire was conducted among patients aged 18 years and above, attending the General Outpatient Clinic of Federal Medical Centre, Asaba, Delta State, Nigeria (n= 366). Data were analyzed using SPSS version 26 (Armonk, NY: IBM Corp.). Univariate and bivariate analyses were conducted at an alpha level of significance set at p<0.05.

Results

This study comprised 366 participants, of which 56.28% were willing to take a COVID-19 vaccine if it was made available to them. Generally, it was found that participants aged <40 years (56.90%), females (56.88%), singles (57.32%), and unemployed respondents (67.44%) were more willing to receive the vaccine than others. Those who agreed that COVID-19 was not an invention by man (p=0.031; OR=1.64; 95% CI=1.05, 2.58) were more likely to receive a vaccine. Suspicion of the government's intentions about COVID-19 was a perceived barrier by participants to accepting a vaccine.

Conclusion

Our findings represent one of the few estimates of the acceptability of the COVID-19 vaccine in Nigeria. They can be used to guide the planning and development of future public health efforts, increasing the awareness of the COVID-19 vaccine, its acceptability, and its uptake.

## Introduction

Coronavirus disease 2019 (COVID-19) is a respiratory disease caused by the new coronavirus “SARS-CoV-2” [1]. The COVID-19 pandemic has resulted in unimaginable harm to the life, health, and economy of nations and individuals, as the World Bank estimated that this pandemic-induced new poor were between 119 and 124 million in 2020 [2]. Along with hygienic and behavioral changes, vaccination provides the best hope for a permanent solution [3].

Researchers across the globe have worked tirelessly to develop COVID-19 vaccines. In Nigeria, the first batch of Oxford-AstraZeneca COVID-19 vaccine of about 3.94 million doses arrived in the country on March 2, 2021 [4], with a total of 1,697,243 Nigerians having received the vaccine as of May 10, 2021 [5]. However, having the best vaccines will not protect individuals from future infections, if people refuse to accept and use the vaccines.

Concerns about vaccine hesitancy are growing worldwide [5]. In fact, in 2019, the World Health Organization (WHO) identified vaccine hesitancy as one of the top 10 global health threats [6]. In many countries, vaccine hesitancy and misinformation present substantial obstacles to achieving coverage and community immunity [7,8]. In Nigeria, vaccination generally has been on the decline since its record high of 81.5% in 1992, with only 25% of children getting the full dose of diphtheria-pertussis-tetanus (DPT) vaccine by the age of one year according to the 2005 UNICEF progress report [9].

In 2015, the WHO Strategic Advisory Group of Experts on Immunization defined vaccine hesitancy as a "delay in acceptance or refusal of vaccination despite the availability of vaccination services" [10]. In a study done by Lazarus et al. carried out in 19 countries, positive responses to COVID-19 vaccines when available ranged from 88.6% responders in China to 54.9% responders in Russia [11]. This was not so different from a study done in Nigeria by Adebisi et al., where 74% showed a willingness to take the vaccine when available [12].

While most of the available studies on acceptance of COVID-19 vaccine were done prior to the development of a vaccine, this study aimed to gauge the current level of willingness to receive the now available COVID-19 vaccines in Nigeria and to ascertain factors influencing its acceptance. The study also aimed to determine the knowledge of COVID-19 and its vaccine as a preventive tool for COVID-19 infection among patients attending the General Outpatient Clinic of Federal Medical Centre, Asaba, and to determine the perceived barriers to receiving the COVID-19 vaccine among these patients.

## Materials and methods

Study site

This study was carried out on patients attending the General Outpatient Clinic of Federal Medical Centre, Asaba, Delta State, Nigeria. Asaba is the capital city of Delta State in Southern Nigeria [13]. The predominant occupation of people in Asaba is civil service [13]. Federal Medical Centre, Asaba, is a tertiary health institution which at the time of this study has a bed capacity of 320, serving essentially patients within the state as well as those from neighboring states mainly Anambra and Edo. It runs a general outpatient clinic (GOPC) every day of the week. The GOPC serves as an entry point for non-emergent cases visiting the hospital for the first time; these patients are subsequently referred to relevant specialties depending on their mode or reasons of presentation. The GOPC receives about 125 patients daily.

Study design

A cross-sectional study was carried out using paper-based questionnaires assessing patients’ knowledge, risk perception, health beliefs, and COVID-19 vaccine practices. Structured self-administered questionnaires were designed by the authors and distributed to patients attending the GOPC in Federal Medical Centre, Asaba, after obtaining ethical approval and informed written consent from the patient. The questionnaire was adapted to our local settings and the objective of the study following a review of similar studies done in our environment.

Inclusion and exclusion criteria

Patients aged 18 years and above attending the GOPC of Federal Medical Centre, Asaba, between June and August 2021 who consented were included in the study. Those who refused to give their consent and patients attending the GOPC who are not up to 18 years of age were excluded from the study.

Sample size determination

The Raosoft (Seattle, WA: Raosoft, Inc.), an automated sample size calculator was used to ensure the recruitment of a sufﬁciently large number of participants in order to obtain statistically signiﬁcant data, given the chosen margin error of 5%, conﬁdence interval of 95%, and population size of 7,500 individuals (being the approximate number of patients attending the GOPC at a rate of 125 patients per day for three months). This gave a sample size of 366 participants. The attrition rate was already calculated by the Raosoft calculator.

Data collection and analysis

Data for this study were collected by members of the research team comprising resident doctors from various departments in the hospital. The purpose of the study, conﬁdentiality and anonymity of the information requested was explained to the participant in fine detail, and informed written consent was obtained. Adults (18 years of age and above) were asked to ﬁll a questionnaire on basic demographic characteristics and COVID-19 vaccine hesitancy. Data were entered manually into a spreadsheet, transferred, and analyzed using SPSS version 21 (Armonk, NY: IBM Corp.). The statistical test was calculated at a confidence interval of 95%.

## Results

Sociodemographic characteristics of the participants

Table 1 shows the sociodemographic characteristics of the participants. A total of 366 participants completed the questionnaire. The majority of the participants are 20-29 years (70.8%) of age and are Christians (95.9%). Around half of the participants were female (59.6%) Igbos ethnicity (53.3%), single (66.4%), and employed by the government (47.8%).

**Table 1:** Sociodemographic characteristics of participants (n=366).

Variable		Frequency (%)
1. Age (years)	20-29	259 (70.8)
	30-39	31 (8.5)
	40-49	60 (16.4)
	50-59	16 (4.4)
2. Gender	Female	218 (59.6)
	Male	148 (40.4)
3. Ethnicity	Yoruba	73 (19.9)
	Igbo	195 (53.3)
	Hausa	0
	Others	98 (26.8)
4. Religion	Christianity	351 (95.9)
	Islam	6 (1.6)
	African traditional religion	9 (2.5)
5. Marital status	Single	243 (66.4)
	Married	120 (32.8)
	Divorced	3 (0.8)
6. Occupation	Government	175 (47.8)
	Private	53 (14.5)
	Self-employed	95 (26.0)
	Unemployed	43 (11.7)

Knowledge of COVID-19 and the types of COVID-19 vaccines known

**Figure 1:**
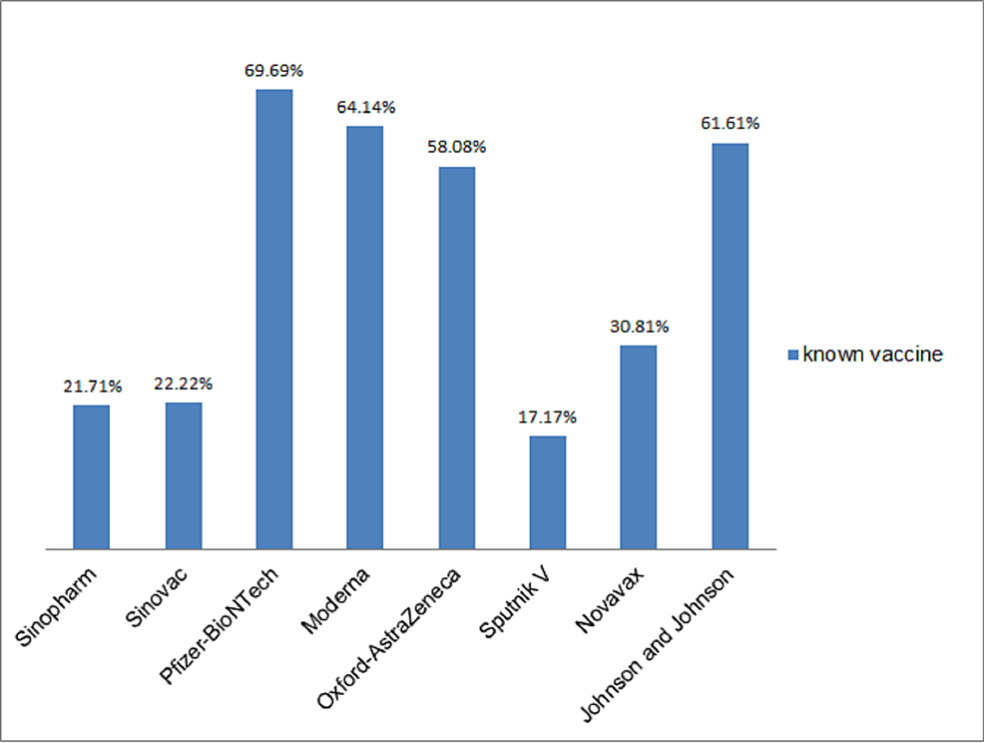
Knowledge of the different types of COVID-19 vaccine (n=198). Only 198 out of the 366 participants agreed they knew at least one type of COVID-19 vaccine. Multiple responses were gotten from these 198 participants. COVID-19: coronavirus disease 2019

Table 2 shows the level of knowledge of COVID-19 among participants, the majority (86.3%) of the participants agreed that COVID-19 causes severe disease and death, and they identified wearing facemasks (95.9%), social distancing (91.5%), and hand hygiene (91.3%) as major ways of preventing COVID-19, only 66.7% of study participants agreed that a vaccine can prevent COVID-19. Information about the disease was gotten mainly from electronic media (77.6%) and social media (74.0%). Slightly more than half (54.1%; n=198) of the participants knew at least a type of the COVID-19 vaccine and Pfizer-BioNTech, followed by Moderna, Johnson & Johnson, and Oxford-AstraZeneca were the vaccines identified (Figure 1).

**Table 2:** Knowledge about COVID-19, its prevention, and COVID-19 vaccine among participants (n=366). Multiple responses were gotten from study participants. COVID-19: coronavirus disease 2019

Variable		Frequency (%)
1. Knowledge of COVID-19	COVID-19 is not real	41 (11.2)
	COVID-19 causes severe disease and death	316 (86.3)
	COVID-19 is an invention by man	118 (32.2)
	Not sure of what to believe about the virus	56 (15.3)
2. How can COVID-19 be prevented?	Social distancing	335 (91.5)
	Hand hygiene	334 (91.3)
	Wearing facemasks	351 (95.9)
	Vaccines	244 (66.7)
	Local herbs	51 (13.9)
	Taking supplements	131 (35.8)
	Some specific medicine	108 (29.5)
3. Source of information about COVID-19	Print media (newspapers, magazines)	204 (55.7)
	Electronic media (TV, radio)	284 (77.6)
	Medical journals/literature	204 (55.7)
	Government, international, and other health authorities	230 (62.8)
	Social media (Facebook, Twitter, Instagram, Whatsapp)	271 (74.0)
	Friends/colleagues	187 (51.1)
4. Knowledge of different types of COVID-19 vaccine	Yes	198 (54.1%)
	No	168 (45.9%)

Factors influencing willingness to receive the COVID-19 vaccination

In this study, only 206 out of the 366 participants (56.28%) said they are willing to take a COVID-19 vaccine. Generally, it was found that participants <40 years (56.90%), females (56.88%), those not married (57.32%), and unemployed (67.44%) were more willing to receive a vaccine (Table 3).

**Table 3:** The sociodemographic characteristics of participants and their willingness to take the COVID-19 vaccine. χ2: chi-square value; ATR: African traditional religion; COVID-19: coronavirus disease 2019

Variable		Willingness to take the COVID-19 vaccine		χ2	p-Value
		Yes (%)	No (%)		
1. Age	<40 years	165 (56.90%)	125 (43.10%)	0.213	0.645
	≥40 years	41 (53.95%)	35 (46.05%)		
2. Gender	Female	124 (56.88%)	94 (43.12%)	0.078	0.780
	Male	82 (55.41%)	66 (44.59%)		
3. Ethnicity	Yoruba	45 (61.64%)	28 (38.36%)	1.278	0.528
	Igbo	109 (55.90%)	86 (44.10%)		
	Others	52 (53.06%)	46 (46.94%)		
4. Religion	Christianity	195 (55.56%)	156 (44.44%)	1.848	0.174
	Islam/ATR	11 (73.33%)	4 (26.66%)		
5. Marital status	Not married	141(57.32%)	105 (42.68%)	0.325	0.568
	Married	65 (54.17%)	55 (45.83%)		
6. Employment status	Employed	177 (54.80%)	146 (45.20%)	2.465	0.116
	Unemployed	29 (67.44%)	14 (32.56%)		

Perception and acceptance level of participants

Using logistic regression analysis, the desire to accept the COVID-19 vaccine was linked to one variable - the virus is an invention by man (Table 4). When comparing the variable as mentioned earlier, it was found that those who disagreed that COVID-19 was an invention by man were more likely to receive a vaccine (p=0.031; OR=1.64; 95% CI=1.05, 2.58). This means that those who said COVID-19 is not an invention by man were 1.64 times more likely to accept COVID-19 vaccination than those who agreed. No significant difference was found for COVID-19 is not real and COVID-19 causes severe disease and death.

**Table 4:** Association between the perception of COVID-19 and the willingness to take the COVID-19 vaccine. *P-value is significant. OR: odds ratio; CI: confidence interval; COVID-19: coronavirus disease 2019

Perception		Willingness to take the COVID-19 vaccine		p-Value	OR	95% CI
		Yes, 206 (%)	No, 160 (%)			
1. Is COVID-19 real?	Yes	25 (12.14%)	16 (10.00%)	0.520	1.24	1
	No	181 (87.86%)	144 (90.00%)			0.64 -2.42
2. It is an invention by man	Yes	76 (36.89%)	42 (26.25%)	0.031*	1.64	1
	No	130 (63.11%)	118 (73.75%)			1.05-2.58
3. It causes severe disease or death	Yes	177 (85.92%)	139 (86.88%)	0.792	0.92	1
	No	29 (14.08%)	21 (13.13%)			0.50-1.69

Perceived barriers by participants towards receiving the COVID-19 vaccination

Using logistic regression analysis, the desire to accept the COVID-19 vaccine was linked to one variable - suspicion of the government's intentions about COVID-19 and the vaccines (Table 5). When comparing this variable, it was found that those who disagreed that the vaccine was embedded with a microchip were more likely to receive a vaccine (p=0.015; OR=0.38; 95% CI=0.20, 0.75). There is also a 2.5 times more chance of taking the vaccine in those who refused to see the vaccine as related to 5G and that it can be used to control someone than in those who agreed to it (p=0.007; OR=2.50; 95% CI=1.29, 4.85). No significant difference was found in the side effects of the vaccine, religious beliefs, and cultural beliefs.

**Table 5:** Association between perceived barriers and willingness to take the COVID-19 vaccine (n=366). *P-value is significant. OR: odds ratio; CI: confidence interval; COVID-19: coronavirus disease 2019

Perceived barriers			Willingness to take the COVID-19 vaccine		p-Value		OR			95% CI		
			Yes, 206 (%)	No, 160 (%)								
1. Side effects of the vaccine												
a. Its possible side effects interference with activities	Yes	131 (63.59%)		105 (65.63%)		0.687		0.92			1	
	No	75 (36.41%)		55 (34.37%)							0.59-1.41	
b. No compensation for possible side effects	Yes	147 (71.36%)		117 (73.13%)		0.709		0.916			1	
	No	59 (28.64%)		43 (26.87%)							0.58-1.45	
2. Suspicion about the intentions of the COVID-19 vaccine												
a. No trust in the government’s approach	Yes	131 (63.59%)		105 (65.63%)		0.687			0.92			1
	No	75 (36.41%)		55 (34.37%)								0.59-1.41
b. The vaccine is embedded with a microchip	Yes	55 (21.15%)		32 (20.00%)		0.015*			0.38			1
	No	151 (78.85%)		128 (80.00%)								0.20-0.75
c. The vaccine is related to 5G and can be used to control someone	Yes	40 (19.42%)		41 (25.63%)		0.007*			2.50			1
	No	166 (80.58%)		119 (74.37%)								1.29-4.85
3. Religious reasons	Yes	51 (24.76%)		40 (25.00%)		0.958			1.01			1
	No	155 (75.24%)		120 (75.00%)								0.62-1.63
4. Cultural reasons	Yes	11 (5.34%)		7 (4.38%)		0.809			1.23			1
	No	195 (94.66%)		153 (95.63%)								0.47-3.26

## Discussion

According to the World Health Organization, vaccine hesitancy is defined as the delay in the acceptance or blunt refusal of vaccines and has been identified as a growing trend in global health [14]. We found that nearly 57% of our study participants would be willing to get a COVID-19 vaccine if one becomes available. Our finding is similar to a study done in Kaduna, a Northern state in Nigeria, in which 55% of the study participants were willing to take the COVID-19 vaccine [15]. However, data from a study in Abuja, also in the Northern part of the country, recently made available online, showed a high hesitancy for the COVID-19 vaccine, of which only 23% of study participants were willing to take the vaccine [16]. The various patterns of acceptance in different parts of the country may be related to regional factors. Though the rise of insurgency has made the immunization situation worse in the northern regions, religious leaders are also said to share hesitation and mistrust against the idea that vaccination is a Western scheme to sterilize people [17]. The intricate relationship between religion and the interaction with psychosocial factors raises the country's prevalence of vaccination hesitancy and may provide an explanation for the observed trends in vaccination coverage as reported by the country. However, the vaccine hesitancy seen in the country is low than in other West African countries. A survey done in five West African countries showed a high hesitancy for the COVID-19 vaccine. This high hesitancy was seen more in Senegal (79%), Liberia (66%), and Niger (58%), while Benin (48%) and Togo (48%) were evenly divided on whether they are likely to get vaccinated or not [18]. Studies conducted in other parts of the world gave more acceptability to the COVID-19 vaccine when compared to our findings. In the United States, it ranges from 50% to 75% [19-21], France (62%), Denmark and the United Kingdom (80%) [22], and in India, it is stated at nearly 81% [23].

Our findings also showed that younger individuals (<40 years) were more willing (56.90%) to take the vaccine compared to the older respondents (53.95%), this is in contrast to the study done by Adedeji-Adenola et al., where it was seen that respondents within the age of 40-69 years were more willing to take the vaccine [24]. This disparity may be due to the prominent use of social media channels seen more in the younger population and the widespread information regarding the benefits of the COVID-19 vaccine which has been effectively propagated using social media platforms. The unemployed were also more willing (67.44%) to get vaccinated than the employed respondents (54.80%), this may be due to the perceived side effects of the vaccine which may hamper productivity in the workplace.

A total of 86.3% of our study participants recognized that COVID-19 is real and can cause severe disease and death, this level of knowledge among the participants can be attributed to the massive scale of information about COVID-19 that has been anchored by government and non-governmental institutions, using various media agencies, including the electronic media (television and radio), social media, print, medical journals, and literature with the major source of information for our participants being the electronic (77.6%) and social (74.0%) media. This level of awareness is similar to the study done by Owhonda et al. as it showed that the knowledge of COVID-19 was prominent within the population in the South-South region of Nigeria [25].

We also inquired about the different preventive measures in curtailing the COVID-19 pandemic, and our study revealed that although social distancing, wearing of facemasks, and hand hygiene were vastly regarded as preventive measures, the COVID-19 vaccine was not given a high preference, with the vaccine receiving 66.7% response as compared to 95.9%, 91.5%, and 91.3% for wearing of facemasks, social distancing, and hand hygiene, respectively. This could be due to the early sensitization drive during the initial phases of the pandemic where the emphasis was placed on these three preventive measures. Also, the use of the COVID-19 vaccine started in Nigeria a year after the first case of the disease was seen in the country [24]. However, before the roll-out of the COVID-19 vaccination exercise, the Nigerian government commenced a rigorous and well-reaching awareness campaign in collaboration with UNICEF and other agencies such as the National Primary Health Care Development Agency (NPHCDA) so as to reduce obstacles related to vaccine hesitancy [24]. Sensitization programs were held with stakeholders in the private sector, healthcare workers, youth leaders, religious bodies, community heads, and traditional leaders in order to raise the momentum [24]. The president and other political figures took turns to take the vaccines live on national television, this was done to allay the fears of vaccine safety and to promote confidence in the vaccine.

Our study showed that the study participants had good knowledge about the different types of COVID-19 vaccines with Pfizer-BioNTech, Moderna, and Johnson & Johnson having more responses than other vaccines as they gathered 69.69%, 64.14%, 61.61%, respectively from the participants, this is striking as the Oxford-AstraZeneca vaccine which had 58.08% of the response from the participants was the first vaccine to be used in the country for the initial phase of the COVID-19 vaccination campaign, this was because of its benefits in its storage as it required warmer temperature requirements compared to others, this benefit is as a result of the unreliable power supply and the need for ultra-cold temperature for storage of other vaccines [26]. Pfizer-BioNTech, Moderna, and Johnson & Johnson COVID-19 vaccines were later introduced into the country from donations from the US government and the African Union Commission [27].

Findings from our study revealed that the majority of the respondents did not have misconceptions about COVID-19, as they dispelled the notion that COVID-19 was man-made and the disease was not real, however, even with the knowledge of the respondents about the disease, we discovered some barriers as the reasons for the hesitancy, these include the perceived side effects of the vaccine interfering with their daily activities and the lack of trust in the government’s approach. Studies done in African countries have shown similar reasons for the COVID-19 vaccine hesitancy seen in our study [28-30], as side effects of the vaccine have been a major contributing factor in dissuading the population from taking the vaccine [28-30]. Government mistrust is also a leading factor, as this has been reported in multiple studies [31-33]. Other factors which have also played in COVID-19 vaccine hesitancy include the effectiveness of the vaccine [3,34], social influence [11], vaccine mandate [35], conspiracy beliefs [3,11], as a small percentage of respondents in this study believed that the COVID-19 had a microchip within it and some believed that it was related to 5G, which could be used to control them. These conspiracy beliefs and insufficient information about the COVID-19 vaccine have been shown to be important factors limiting the successful implementation of the COVID-19 vaccination campaign in conjunction with the safety profile of the vaccines, mistrust in government policies, and poorly equipped health systems [11,31].

As seen from this study, 43.7% of the respondents were hesitant to receive the COVID-19 vaccine, and having seen some of the reasons from the respondents, it is clear that strategies are needed to mitigate this level of vaccine hesitancy. It has been suggested that communication strategies are key to ensuring trust in the vaccines [36], coupled with vaccine delivery techniques, as this is meant to make the process transparent, honest, accurate, truthful, multimodal, and frequent, in partnership with the health workers and community in an inclusive manner [36]. Afolabi et al. affirmed this suggestion, as the need for community mobilization was stressed in order to refute false reports on the COVID-19 vaccine and ensure health education on the benefits of the vaccine [37].

The limitations of this study include the following: (1) its cross-sectional design, which was based on self-reporting and is therefore subject to information bias; (2) the study participants are patients of a particular department in a tertiary hospital in Nigeria, this limitation should be considered in interpreting and applying the results of the current study to the Nigerian populace; (3) our study assessed vaccine acceptability under the condition that the vaccine was free, and acceptability might be lower if there would be out-of-pocket costs associated with the vaccine; (4) the study has low power, hence more studies are needed to draw meaningful conclusions for future interpretation; (5) It is a self-administered questionnaire-based study and may affect the response of respondents who are not educated. However, the logistic regression and the large sample size make this a relevant piece of research and thus is the strength of the study.

## Conclusions

The COVID-19 vaccine is a key public health strategy for reducing the overall disease burden due to COVID-19. Our study provides insight into the acceptability of the COVID-19 vaccine, with results indicating that a little more than half of the study population will be willing to get vaccinated. This has been attributed to several demographic characteristics, as well as the key role that the government, healthcare providers, and perceived beliefs play in the acceptability of a COVID-19 vaccine. Our finding represents one of the few estimates of the acceptability of a COVID-19 vaccine in Nigeria and can be used to guide the planning and development of future public health efforts significantly increasing awareness of the COVID-19 vaccine, its acceptability, and its uptake. Findings from this study will serve as a guide to government institutions and stakeholders in addressing the factors limiting the acceptance of the vaccine, improve the perception of the population towards the vaccine and ensure the successful vaccination program with the aim of limiting the burden of the COVID-19 disease to the nearest minimum.
